# Perspectives of Patients and Therapists on Social Media and Digital Data Use in Mental Health Therapy: Thematic Analysis

**DOI:** 10.2196/32103

**Published:** 2022-07-07

**Authors:** Lauren Southwick, Rebecca Suh, Elissa Kranzler, Megan Bradley, Raina M Merchant

**Affiliations:** 1 University of Pennsylvania Philadelphia, PA United States; 2 Fors Marsh Group Washington DC, DC United States; 3 Department of Emergency Medicine Perelman School of Medicine Philadelphia, PA United States

**Keywords:** social media, digital health, digital data, mental health therapy, mobile phone

## Abstract

**Background:**

Incorporating insights from social media into the patient-provider encounter is increasingly being explored in health care settings. Less is known about the utility of these data in mental health therapy.

**Objective:**

This study aims to prospectively investigate and characterize how social media and digital data are used in mental health therapy from both the patient and mental health therapist perspective.

**Methods:**

Patients enrolled in mental health therapy and mental health therapists were interviewed using a semistructured interview guide. All interviews were transcribed and coded using a deductive framework analysis. Themes and subthemes were identified. Participants completed a sociodemographic survey, while mental health therapists also completed a behavioral norms and elicitation survey.

**Results:**

Seventeen participants, that is, 8 (48%) mental health therapists and 9 (52%) patients were interviewed. Overall, participants identified 4 themes and 9 subthemes. Themes were current data collection practices, social media and digital data in therapy, advantages of social media and digital data in therapy, and disadvantages of social media and digital data in therapy. Most subthemes were related to the advantages and disadvantages of incorporating digital data in mental health therapy. Advantage subthemes included convenience, objective, builds rapport, and user-friendliness while disadvantage subthemes were nonreflective, ethically ambiguous, and nongeneralizable. The mental health therapists' behavioral norms and elicitation survey found that injunctive and descriptive normative beliefs mapped onto 2 advantage subthemes: convenience and objectivity.

**Conclusions:**

This qualitative pilot study established the advantages and disadvantages of social media and digital data use in mental health therapy. Patients and therapists highlighted similar concerns and uses. This study indicated that overall, both patients and therapists are interested in and are comfortable to use and discuss social media and digital data in mental health therapy.

## Introduction

### Background

Adults in the United States are frequent users of social media platforms such as Facebook, Instagram, and Twitter [1,2]. Such platforms have a profound impact on everyday life and provide new opportunities for understanding behavioral, social, and environmental determinants of mental health and well-being [3-5]. Social media data (eg, Facebook wall posts) and digital data (eg, search engine use, step data, smartphone metadata) are increasingly used to support patient mental health care [6]. Previous mental health research has demonstrated that social media data can reveal and predict risk for mental health conditions such as depression, loneliness, suicide ideation, posttraumatic stress, schizophrenia, and bipolar disorder [6-12]. Furthermore, prior research has demonstrated that data from these digital platforms can provide critical information not readily attained through in-person or remote health care encounters to help therapists identify, address, or discuss mental health concerns [3,13,14].

In response to these findings, several researchers are increasingly capturing how and how often therapists incorporate social media data into mental health therapy and treatment. For example, Fisher and Appelbaum [15] report that ﻿some mental health clinicians incorporate parts of their patients’ Facebook feeds in their care delivery, whereas Hobbs and colleagues [13] found that nearly two-thirds of outpatient psychotherapists report viewing at least one patient’s social or electronic media (such as email messages, SMS text messaging, and other messaging apps) as part of psychotherapy. Hobbs et al [13] also report that the psychotherapists who access their patients’ electronic or social media data indicated that it improved their ability to provide effective treatment. These findings underscore the utility of social media and digital data in mental health therapy. These examples reflect the growing body of research on the mental health care therapist experience using social media data in mental health therapy; however, research on the patient perspective remains limited. Accordingly, research exploring both patient and therapist perspectives on the use of social media data in mental health therapy is warranted.

### Objective

This qualitative study aimed to provide new knowledge on mental health patient and therapist perspectives regarding the use of social media data in mental health therapy. The aims of this study were to (1) explore patients’ and therapists’ current use(s) of social media data in mental health therapy and (2) identify the advantages and disadvantages of sharing social media data in mental health therapy.

## Methods

### Recruitment

Individuals in mental health therapy (referred to as patients) were recruited through a clinical research registry at a large academic institution (a private university and medical center in the northeastern region of the United States) from March to May 2018. Thirteen applicants expressed interest from the registry, of which 9 were available for an in-person interview. Patient inclusion criteria included that they attended mental health therapy for anxiety or depression and were aged 18 years and older. Patients completed informed consent and received a US $20 gift card as compensation for their study participation. Mental health therapists (referred to as therapists) were recruited at the same large academic institution through behavioral health research consortiums and word-of-mouth sampling (approximately 50 received an email invitation) from October to December 2018. Twelve therapists expressed interest and 8 were available for an in-person interview. Therapist inclusion criteria included that they provided behavioral health care and worked with clients aged 18 years and older with depression or anxiety disorder(s). The therapists completed informed consent and received a US $100 gift card as compensation for study participation. All participants were willing to complete an in-person interview at a large academic hospital, which was held in a secure private room and lasted for 50-70 minutes.

### Qualitative Interview

Two semistructured interview guides were constructed based on a review of the published literature on social media data in therapy and co-design techniques [15,16]. The interview guide was structured as follows: introduction, discussion of interview expectations, current use of data in therapy, experience with social media data (eg, Facebook, Instagram, Twitter) in therapy, and advantages and disadvantages of incorporating social media data in therapy. Interview questions were tailored to separately address the patient and therapist experiences. All interviews were conducted by 2 team members. One individual (a cisgender female researcher with >10 years of experience in qualitative interviewing) acted as the lead facilitator, while the other interviewer (a cisgender female service designer with 5 years of experience) took notes and contributed to probing and follow-up questions. Prompts were included, where appropriate, to elicit participant elaboration about each topic. The full interview guide is available from the authors upon request.

### Survey

Patients completed a brief in-person survey at the end of the interview. The survey assessed sociodemographic characteristics such as gender, race/ethnicity, highest level of education, and the Social Media Use Questionnaire (SMUQ) [17]. The SMUQ assesses problematic use of social media and comprises 9 items (eg, “I feel anxious when I am not able to check my social network account”), with response options on a 5-point Likert scale from “Never” to “Always.” All items were averaged into a scale for which higher scores corresponded with excessive social media use.

At 24 hours after the interview, therapists received a “thank you” email and a link to a web-based survey, programmed in Qualtrics software. The survey was intended to elicit participants’ beliefs pertinent to using data about patients’ social media use in therapy sessions, given little existing research on social media in mental health therapy. Survey questions were drawn from established procedures for elicitation studies [18]. These open-ended questions elicited beliefs about using social media data in therapy sessions in the next month. Types of beliefs elicited included behavioral beliefs (ie, potential benefits or drawbacks of using social media data in therapy sessions), injunctive normative beliefs (ie, individuals or groups of people who may or may not approve of using social media data in therapy sessions), descriptive normative beliefs (ie, individuals or groups of people who may or may not use social media data in therapy sessions), and control beliefs (ie, circumstances that may help or hinder the use of social media data in therapy sessions). The survey also assessed the sociodemographic characteristics of the participants, including gender, race, ethnicity, highest level of education, and work environment characteristics (eg, type of practice, caseload).

### Ethics Approval

This study protocol was reviewed and approved by the University of Pennsylvania Institutional Review Board (protocol 831246). 

### Analysis

#### Qualitative Interviews

Therapist interviews were audio recorded and transcribed verbatim. Patient interviews were not recorded; a research assistant observed the interviews and took detailed notes and collected quotes. After multiple readings, the transcripts and interview notes were then coded by authors LS and RS. The coders created a codebook using the interview guide as themes, whereas subthemes emerged from the transcripts. The transcripts and interview notes were then analyzed using a deductive framework analysis [19] and coded according to the 6 stages of framework analysis: familiarization, identifying a thematic framework, indexing, charting, mapping, and interpretation. Identified subthemes were deduced from the coded passages and placed into separate coded charts. The authors then independently reviewed the charts for consistency and agreement. The coders met regularly to resolve disagreements for any theme or subtheme by consensus following discussion. The codebook is available from the authors upon request.

#### Survey Findings

All quantitative analyses (ie, descriptive statistics) were conducted in a Microsoft application (Microsoft Excel, version 16.58).

## Results

### Study Sample

The sociodemographic characteristics of the patients and therapists are provided in Table 1. Patients were mostly female (6/9, 66%) and non-Hispanic White (6/9, 66%). Patients were aged 22-60 years; half reported having completed college while the other half completed graduate school. According to the SMUQ, all patients reported using social media platforms (eg, Facebook, Instagram) more than 3 times a day and exhibited nonproblematic social media use (mean score 22, range 9-32). All therapists were females and non-Hispanic White, with a mean age of 37 years. Most reported that they work in a hospital/medical setting and practice cognitive behavioral therapy (CBT). However, therapists were at various stages in their careers, ranging from graduate student to psychologist or associate professor (Table 1).

**Table 1:** Sociodemographic characteristics of the patients and therapists.

Sociodemographic variables		Patients (n=9)		Therapists (n=8)	
		Frequency/mean	Range	Frequency/mean	Range
Age (years)		34	22-60	37	29-43
Female		6	N/A^a^	8	N/A
**Ethnicity/race**					
	White	6	N/A	8	N/A
	Black	1	N/A	0	N/A
	Asian	1	N/A	0	N/A
	Non-Hispanic, Latinx	1	N/A	8	N/A
**Highest level of education**					
	College graduate	5	N/A	0	N/A
	More than college graduate (eg, master’s, doctoral degree)	4	N/A	8	N/A
Social media use questionnaire		22	9-32	N/A	N/A
Cognitive behavioral therapy orientation		N/A	N/A	8	N/A
Clinical experience (years)		N/A	N/A	5.3	2-9
**Clinical setting**					
	Community outpatient	N/A	N/A	1	N/A
	Hospital	N/A	N/A	4	N/A
	Private practice	N/A	N/A	3	N/A
Caseload (clients)		N/A	N/A	N/A	5-40
Prep time prior to session (minutes)		N/A	N/A	N/A	3-30

^a^N/A: not applicable.

### Thematic Areas

The interviews captured patients’ and therapists’ social media use in mental health therapy. The interviews identified the advantages and disadvantages of sharing social media data in mental health therapy and highlighted the contextual and logistical considerations to incorporating these new data. The interviews were structured on the following themes: (1) current data use in therapy, (2) experience with social media in therapy, (3) advantages of social media in therapy, and (4) disadvantages of social media in therapy (Table 2).

**Table 2:** Emerging interview subthemes with illustrative quote(s).

Themes, subthemes			Illustrative quote(s)		
			Therapist		Patient
**Current data use in therapy**					
	Applications	…*I think it is a great idea because if you get the notification from the app and it is kind of fun, it’s visually enticing, and people are more likely to do it. And then some of them have the built-in mindfulness tracking.*		…*I use the eMoods app to track my triggers and love the biometrics information. It tracks a lot things like eating vegetables, how much bread I eat, or if I went outside. I show my therapist it. eMoods doesn’t feel like it’s judgmental because I am creating the field(s).*	
	Surveys	…*Implement surveys during treatment to see change over time in symptoms or quality for life.*		…*Surveys are infrequent, I attend a self-determined care model.*	
**Experience with social media in therapy**					
	Patient-initiated	…*The patient brought up Snapchat and showed conversation around the video…It was sharing a sexting video with someone they were interested in and were having anxious thoughts about having done that and the individual saved/downloaded the video.*		…*I am comfortable sharing social media because it’s already out there, and everyone puts their business out on social media.* * …I am willing to share [with my therapist] if I can explain and provide context.*	
	Provider-initiated	…*There’s a lot of videos on YouTube to use for exposures. So…if I have somebody that comes in with like a serious vomit fear or emetophobia or fear of like, could be anything like scary movies or clowns. We just go on YouTube and look stuff up.*		…*I trust my instincts [on what to share].*	
**Advantages of incorporating social media in therapy**					
	Convenience	…*I would also love for there to be more technology where the person doesn’t have to enter anything themselves. That’s why the Fitbit is nice or this sleep app where you basically put under your pillow (and) it tracks your sleep… because requiring them to do any work when they’re already depressed and anxious [and it can be] a huge burden.*		…*It would be nice to look back [at previous posts].*	
	Objective	…*I think sleep data could be really useful… Like I’ve had a lot of patients who tell me that they sleep for like 3 hours and I’m like that’s not possible every single night. I think this could be useful.*		…*It would help flag my memory… keep me on track.*	
	Builds rapport	…*I don’t think it derails anything… mostly it’s very relevant and helpful. They’re willing to share, and I think it builds rapport. Often times it helps me to like really understand what they’re talking about.*		…*We talk about [text messages] as a “how are you?”*	
	User-friendly	…*It’s nice that it graphs it for you…I would use it in the beginning of session when I’m asking how they are feeling right now and how they feel how the last month has been to sort of see if their self-report in the moment lines up.*		…*If therapy is holistic having this data might not be bad.*	
**Disadvantages of incorporating social media in therapy**					
	Nonreflective	…*I think that people sometimes present very differently in therapy than they might on social media. If I’m thinking of somebody who is super depressed or maybe [have] chronic mental health issues, I think that that person probably doesn’t post a lot on social media about their depression.*		…*I trust my instincts on what to share.* * …I want to disclose what I need to [in each session]… maybe it’s my eating disorder [that] has been more on my mind, not my substance abuse.*	
	Ethically ambiguous	…*For them to know that I have access to everything they post could make them feel pretty watched. They don’t have any privacy because anything they do online the therapist will see it and will pull it up on their portal and judge them for it.*		…*My phone is so personal and feels like an invasion of privacy. It feels too “big brother.”* * …I am not confident in accuracy of an algorithm.*	
	Nongeneralizable	…*I think there are different social media personalities. There are some people who are very explicitly like “I don’t talk about politics on Facebook.” And other people who use it as their diary where they are posting every thought. And then people who don’t post very much because they’re more private.*		…*I [would like to] annotate them (ie, text message or social media post) because things are left out.* * …I don’t [want to be] judged by my digital data.*	

#### Current Data Use in Therapy

When asked about current data collection practices, both patients and therapists noted using mental health and well-being apps in their sessions. Apps were mainly used during their practice to augment mental health services. For example, a patient reported using an app designed for patients with bipolar disorders I and II, depression, posttraumatic stress disorder, and anxiety disorder to keep track of symptoms and triggers (Table 2). However, several therapists expressed concern with selecting the “right” app, whereas others noted security and confidentiality issues for their patients. One therapist said, “[My patient] didn’t want to put the app in her phone because her friends look at her phone and she thought that they would ask about it” (Participant #1, female, aged 42 years). In addition to using apps, therapists reported that they routinely collect patient-reported outcome data. As a common practice of CBT, validated surveys such as Beck’s Depression Inventory, Patient Health Questionnaire 9-item, or Generalized Anxiety Disorder 7-item scales are collected at every therapy visit to track progress. Patients in this sample did not report completing validated surveys at their therapy sessions.

#### Experience Discussing Social Media in Therapy

The patients interviewed reported sharing and discussing social media data with their therapist. Most patients reported sharing or discussing Instagram posts (n=10), Facebook posts or statuses (n=8), tweets (ie, Twitter posts) (n=3), and YouTube videos (n=3). One patient noted, “I show my therapist my Facebook, it’s evidence of my life; yes, I have friends, I exist” (Participant #3, genderqueer, aged 24 years).

Approximately half of the patients reported that they have directly shown or summarized social media posts to their therapist in the last month. Therapists noted similar interactions; one elaborated as follows, “So for [instance] a patient that is dating and wants to show me some of the people that she’s talking about… she’ll show me on social media. Or they want to show me what they post, a story that’s going, something that they are involved with, like a family member or friends” (Participant #2, female, aged 35 years). Once social media data are introduced in therapy sessions, both patients and therapists detailed how social media platforms such as Facebook or YouTube can also be used as homework or in exposure therapy. For example, 1 patient explained that their therapist would have them write on a friend’s Facebook wall to address their social anxiety and phobia.

#### Advantages of Incorporating Social Media in Therapy

Both patients and therapists highlighted how social media data can be convenient, objective, user-friendly, and allow them to build rapport when used in therapy (Table 2). Both patients and therapists focused on how data are shared, may it be automatic or manual and how the algorithm could select posts as an advantage. Patients indicated a general acceptance of an algorithm selecting their social media posts but emphasized a desire to annotate or provide context to the post. One patient said, “I need to be there with [my therapist] to review” (Participant #5, male, aged 34 years). Therapists highlighted a desire for objective metrics derived from social media posts. They commonly referenced social media’s metadata such as time of post and language used. For example, therapists noted that patients recounting of events may be influenced by recall bias when individuals have a partial account of prior events [20], whereas seeing metadata provides an objective source of information. Both therapists and patients highlighted their comfort with and interest in digital data such as steps walked via a smartphone’s built-in pedometer or screen time metrics.

Social media data are seen to aid discussions and accelerate a patient’s account of events. Both participants and therapists highlighted how they would like to see trends over time. A therapist said, “I’m asking how they are feeling right now and how they feel how the last month has been to sort of see if their self-report in the moment lines up” (Participant #2, female, aged 35 years). Therapists also noted instances when their patient would show pictures of friends or family from a social media platform to add a “*face to the name*,” implying that the use of such data builds rapport, which in turn could enhance the patient-provider communication.

#### Disadvantages of Incorporating Social Media in Therapy

Our results underscored perceived disadvantages of using social media data in therapy. Specifically, it can be nonreflective, nongeneralizable, and its use could be ethically ambiguous. Patients reported a sense of fear and uneasiness “*always*” sharing social media data with their therapist. They noted concerns about being “*watched*” and saw it as “*a little creepy*.” Few therapists expressed concern that it could also elicit a Hawthorne effect, altering one’s behavior due to the awareness of being observed [21]. As 1 therapist noted, “And again, me seeing all of [the] posts patients put up, even if they are agreeing to that…Patients may change [the way] they engage with that social media platform” (Participant #6, female, aged 43 years).

Therapists expressed concern that sharing social media data is nonreflective and would not provide accurate depictions of the patient’s true thoughts and emotions. They highlighted how social media posts are often public-facing accounts of people or events and may not be genuinely authentic. One patient said that “*things are left”* out of posts, whereas therapists highlighted how their patients may have a social media personality.

Several participants raised important questions regarding the security of the data collected, with 1 patient saying, “I want to protect [my] autonomy” (Participant #7, female, aged 29 years). Similarly, a patient highlighted that that they trust their intuition on what social media posts to share with their therapist (Participant #7, female, aged 29 years). Half of the therapists expressed concern regarding the social media platforms’ security policies. Several therapists also indicated that consistently including social media data in their therapy sessions could negatively impact their workflow.

Furthermore, utility of social media data in therapy may hinge on a patient’s age or comfort with technology. Both therapists and patients agreed that including these data would be the most beneficial for younger or more technologically inclined patients. One patient participant noted that social media in their therapy session may not be “beneficial for me [and my treatment goals] but for a younger generation because they post so much” (Participant #6, female, aged 60 years).

### Additional Therapist Beliefs About Social Media Use in Therapy

After the interviews concluded, therapists were asked a series of open-ended survey questions to assess their normative and control beliefs relevant to using patients’ social media data in therapy sessions. See Table 3 for the elicitation questions asked and the illustrative quotes. Therapists were asked to indicate what types of individuals or groups of people would be more or less likely to use social media in therapy (descriptive normative beliefs). Responses included being a younger therapist or patient and a digital native and someone who grew up with technology who might be more technologically inclined. Therapists indicated that their colleagues who would be open to this type of data exchange must be made aware of the social media platform themselves and be oriented toward CBT or other measurement-based care orientations. Therapists reported that individuals or groups who would be less likely to use social media in therapy included those who are older, have limited experience with social media platforms, and do not use measurement-based care. When asked to indicate what circumstances would make it difficult or easy to use social media in therapy (control beliefs), responses underscored the importance of convenience of use for both the therapist and the patient and ease of understanding in the context of therapy.

**Table 3:** Examples of open-ended responses and the corresponding theme categorization for each type of belief elicitation question.

Belief category, elicitation questions, illustrative open-ended responses				Subtheme(s)
**Normative beliefs (injunctive)**				
	**Generally, what types of individuals or groups would approve or think you should use data about clients’ social media use in therapy sessions in the next month? Please list general groups or personas; do not include specific names.**			
		Younger clinicians, clinicians who work with young adults, clinicians who use a measurement-based care framework (ie, track their clients’ progress using measures)	Convenience Objective	
		Younger therapists, data-driven/number-oriented people	Objective	
	**Generally, what types of individuals or groups would disapprove or think you should not use data about clients’ social media use in therapy sessions in the next month? Please list general groups or personas; do not include specific names.**			
		Potentially psychodynamic practitioners, individuals with strong privacy concerns	Ethically ambiguous	
		Individuals who do not use or have social media, clients who may be mistrusting or not have a strong therapeutic rapport with their therapist	Builds rapport Ethically ambiguous	
**Normative beliefs (descriptive)**				
	**Generally, what types of individuals or groups are most likely to use data about clients’ social media use in therapy sessions in the next month? Please list general groups or personas; do not include specific names.**			
		Younger, more number-oriented practitioners	Convenience Objective	
		Clinicians who are familiar with and comfortable using social media, clinicians who treat young adults, clinicians who incorporate technology into their treatments (eg, give measures on a computer or iPad, email, or text their clients)	Provider-initiated	
	**Generally, what types of individuals or groups are least likely use data about clients’ social media use in therapy sessions in the next month? Please list general groups or personas; do not include specific names.**			
		Older clinicians: clinicians in an environment in which it is inconvenient to do so	Convenience	
		Clinicians who do not use social media themselves and may have limited knowledge about how to use it (likely older clinicians), clinicians from orientations that do not emphasize measurement	N/A^a^	
**Control beliefs**				
	**Please list any factors or circumstances that would make it easy or enable you to use data about clients’ social media use in therapy sessions in the next month.**			
		Easy-to-use interface that logs all the information on the patient unobtrusively	User-friendly Convenience	
		Electronic platform, automated data collection and reminders	Convenience	
		Easily downloadable app(s), clear directions on how to use it in sessions with clients	Apps Convenience	
	**Please list any factors or circumstances that would make it difficult or prevent you to use data about clients’ social media use in therapy sessions in the next month.**			
		Lack of perceived need or benefit for a client, privacy concerns, difficult user interface for either myself or my client	Nonreflective	
		If I had to go through several steps to access the data	Convenience	
		A client’s hesitancy or anxiety	Ethically ambiguous	

^a^N/A: not applicable.

## Discussion

### Principal Findings

This qualitative study provides new knowledge on patient and therapist perspectives regarding the use of social media in mental health therapy. The interviews captured patients’ and therapists’ current use(s) of social media in mental health therapy and that both patients and therapists initiated its use and discussion in prior sessions. Of note, the individuals interviewed expressed comfort reviewing and discussing social media data. They identified several advantages and disadvantages of sharing social media data in mental health therapy (Table 2), underscoring the utility of and the potential concerns associated with integrating elements of our digital lives into mental health therapy in a world that increasingly relies on digital technologies.

To our knowledge, this is the first account of patients’ experiences and perspectives on this novel data source. Patients highlighted how social media posts provide objective evidence of their social lives or networks. They discussed previous experiences of self-curating posts and questioned if they want an algorithm selecting social media posts to share. Throughout the interviews, a clear understanding of how the data are generated and shared was of great importance. Previous research has captured individuals’ willingness to share digital data for health research [3,22]. However, researchers have yet to devise a way to provide comprehensive feedback or snapshots to better inform patients, let alone their health care provider.

Despite already using social media data in therapy, both patients and therapists questioned the utility of certain social media platforms. They noted that social media posts may not fully reflect their true thoughts or feelings. This speaks to a growing trend among Instagram users with 2 types of accounts: “finsta” accounts, on which users post less polished photos of themselves and “rinsta” accounts, which include more authentic posts [23]. “Finsta” and “rinsta” accounts highlight how social media posts may not always accurately portray individuals’ experiences. However, there might be clinical relevance if a patient has a “rinsta” account.

The desire for objectivity was noted throughout the interviews. Although the interview questions specifically asked about social media data, therapists discussed the incorporation of objective digital data such as the number of steps taken per day or hours slept per night and highlighted them as variables that can contribute to one’s mental health and well-being. Although our interviews did not specifically capture the use of digital data such as steps and screen time, Di Matteo and colleagues [24] reported general acceptance sharing this type of data through their interviews with new patients referred to a tertiary care mood and anxiety disorder clinic. Since Di Matteo et al’s work [24] and our interviews were both hypothetical scenarios, further research is needed to assess patient and therapist comfort with sharing social media data in the context of therapy sessions.

Lastly, when discussing how to systematically incorporate social media data into sessions, both patients and therapists stressed the importance of person-centered design. As demonstrated by Yoo and colleagues [25], who conducted co-design workshops to build a social media tool for therapists, the data must map onto the end users’ expectations, both in terms of analyses and organization, to avoid being “another layer of noise” [25]. Therapists in this sample questioned how social media data would fit in their workflow (Tables 2 and 3). Despite these concerns, overall, our interviews highlight how sharing social media insights with the patient and therapist could also be clinically relevant and informative. Further work is needed to explore and test how to systematically collect social media and present it to patients and their therapists in a user-friendly format for use in mental health therapy.

### Implications and Recommendations

To our knowledge, this is the first study to capture the patient perspective on their experience sharing and discussing social media data in mental health therapy. The patients interviewed provided critical insights that have yet to be characterized. Our findings uniquely underscore the importance of patient autonomy on what and when to share social media data. As the study sample included mostly female, White, college-educated patients and female, White, and CBT therapists, future research should be conducted with more diverse samples in terms of gender, race/ethnicity, therapeutic orientation, and educational attainment. Additional and special attention is also needed to explore how social media is used and discussed in other cultural contexts.

Our interviews were conducted in 2018 and 2019 prior to the COVID-19 pandemic and may not reflect current norms and beliefs of social media data sharing in the therapeutic encounter. During the COVID-19 pandemic and recovery phrases, there has been a dramatic increase in individuals with depressive and anxiety symptoms seeking mental health care [26]. Further research is warranted to capture how social media data are shared in virtual sessions via videoconference or telephone call. We also recommend additional research on the use of digital data such as smartphone metadata as viable data sources in therapy [27]. The combination of social media and digital data could enhance tailored treatment plans and impact therapeutic alliance, a cooperative working relationship between client and therapist, often seen as an essential aspect of successful therapy [28]. Since previous research found that therapeutic alliance is maintained and even enhanced with the introduction of digital mental health interventions [29,30], further research in this area is needed.

Lastly, there are clear educational, practical, and policy implications. As detailed in the American Psychological Association Guidelines for the Optimal Use of Social Media in Professional Psychological Practice (October 2021) [31] and indicated by our findings, mental health therapists should be encouraged to undergo specific educational training on how to safely and ethically use social media data in therapy. The training should encompass core elements of ethics, informed consent, comfort with technology, social media trends per population segment (eg, age, gender, race, ethnicity, sexual identity, language, culture), and how to integrate these new data sources into their workflow. Clinics and practices would need to reserve additional time, personnel, and technology infrastructure to support this training. Noel and colleagues [11] recommend a technology specialist, a new type of health care worker who identifies and reviews electronic resources that may support a client’s specific recovery goals. Data infrastructure and protections are critically important and require special attention. Furthermore, if social media data were incorporated into therapy and demonstrated to improve patient outcomes and reduce costs to the clinic and patient, national/state policies and insurance companies could modify current plans and coverage.

### Limitations

This study has several limitations. First, the study sample was small and largely homogeneous with respect to sociodemographic data. All participants were recruited from a convenience sample in 1 large metropolitan region in northeastern United States. It is possible that the results from this study do not apply to other population segments or geographic regions. As we advertised for this study online, it is possible that study participants were drawn to the study because of their prior experience with social media in mental health therapy. We were unable to audio record patient participant interviews. As such, our transcripts were not as robust for patient participants as they were for therapist participants. Our interviews were conducted with patients and therapists separately; future research could interview patient and therapist dyads for further insights. With respect to analyses, there are limitations to thematic coding as a methodological approach, such as inferences made from a small study sample size and coding at the phrase level, which may not fully capture the participants’ intended meaning. Furthermore, we were unable to use qualitative data analysis software such as NVivo. Interviews were conducted prior to the COVID-19 pandemic. Accordingly, findings may not fully reflect the current state of mental health delivery in the United States, as most mental health therapy is now delivered virtually via videoconference [32]. Despite these limitations, findings from this pilot study can inform social media use practices and norms in mental health therapy.

### Conclusions

In this study, patient and therapist interviews provide important insights on the current utilization of social media in mental health therapy, including the advantages and disadvantages of social media use in such contexts. Our findings highlight that social media data used in therapy provide convenient, objective information that is user-friendly and can promote rapport between the patient and the therapist. However, the use of social media data in mental health therapy is also perceived as nonreflective, ethically ambiguous, and potentially nongeneralizable. Future research is needed to explore and test how to systematically collect social media and present it to patients and their therapists in a user-friendly format for use in mental health therapy.

## Abbreviations

CBT: cognitive behavioral therapy
SMUQ: social media use questionnaire
